# Elevated C-reactive protein levels at ICU discharge as a predictor of ICU outcome: a retrospective cohort study

**DOI:** 10.1186/s13613-016-0105-0

**Published:** 2016-01-13

**Authors:** S. Sophie Gülcher, Nynke A. Bruins, W. Peter Kingma, E. Christiaan Boerma

**Affiliations:** Department of Intensive Care, Medical Center Leeuwarden, PO Box 888, 8901 BR Leeuwarden, The Netherlands; Raadhuisstraat 18, 9001 AG Grou, The Netherlands

**Keywords:** ICU discharge, Inflammatory markers, Outcome, C-reactive protein, Readmission

## Abstract

**Background:**

Before discharging a patient from the ICU, an adequate patient evaluation is needed to detect individuals as high risk for unfavorable outcome. A pro- or anti-inflammatory status is a potential risk factor for an adverse outcome, and elevated CRP concentrations have shown to correlate with organ failure. Several studies have been performed to evaluate the use of CRP as a marker of post-ICU prognosis. Results are seemingly conflicting, and it is worthwhile to investigate these markers further as CRP is an adequate marker of pro- and anti-inflammatory status of the patient. We aimed to test the hypothesis that elevated CRP levels at ICU discharge are associated with an increased risk of ICU readmission and in-hospital mortality in patients with a prolonged ICU stay.

**Methods:**

A retrospective cohort study was performed in a single-center hospital with an 18-bed mixed medical/surgical ICU. Patients discharged alive from the ICU with at least 48-h ICU length of stay were evaluated. Patients were distributed into two groups: ‘high CRP’ (≥75 mg/L) and ‘low CRP’ (<75 mg/L) at ICU discharge. We assessed the difference in adverse outcome (ICU readmission and/or in-hospital mortality) between these groups.

**Results:**

A total of 998 patients were included. Compared to the ‘low CRP’ group, patients in the ‘high CRP’ group had a higher readmission rate (13.1 vs. 7.4 %; *p* = 0.003). The post-ICU mortality rate in the ‘high CRP’ group and ‘low CRP’ group was 6.9 % and 4.7 %, respectively; *p* = 0.127. Combined readmission and mortality rates were significantly higher in the ‘high CRP’ group in comparison with the ‘low CRP’ group (17.9 vs. 10.1 %; *p* = 0.001). Hospital mortality in patients readmitted to the ICU was significantly higher than in non-readmitted patients (20 vs. 4.3 %; *p* < 0.001). Strikingly, the ‘high CRP’ group had significantly lower APACHE II and SOFA scores at ICU admission compared to the ‘low CRP’ group. This highlights the potential for ICU-acquired risk factors, including CRP.

**Conclusions:**

A high CRP concentration (≥75 mg/L) within 24 h before ICU discharge is associated with an increased risk of adverse outcome post-ICU discharge. However, CRP at discharge represents only a very moderate risk factor and may not be used for individual clinical decision-making.

## Background

To discharge a patient from the intensive care unit (ICU) to another hospital ward is a complex decision-making process [1]. On the one hand, unnecessarily prolonged ICU stay is unwanted. On the other hand, premature discharge from ICU has potential hazards, such as readmission or death [2]. Therefore, an adequate evaluation of the patient before ICU discharge is necessary to detect individuals as high risk for unfavorable outcomes, using clinical predictors and objective parameters, by which readmission to ICU and in-hospital mortality can be reduced [3]. Available scoring systems such as the Acute Physiology and Chronic Health Evaluation (APACHE), the Simplified Acute Physiology Score (SAPS) and the Sequential Organ Failure Assessment (SOFA) scores are not designed to evaluate ICU-discharge risk factors [4–6]. In addition, it is conceivable that attributable risk factors may exist as a result of the course of the disease during ICU stay or individual patient response to disease- or ICU-related insults [7].

A potential risk factor at ICU discharge is the pro- or anti-inflammatory status of the patient. Easily accessible markers of these processes are body temperature, leukocyte count and C-reactive protein (CRP) levels. CRP concentrations correlate with ongoing organ dysfunction and ICU mortality [8]. This marker is routinely measured at ICU and has advantages of simplicity, reproducibility and speed [9].

Several studies have been performed to predict in-hospital mortality and readmission after ICU discharge in relation to signs of inflammation [10–14]. Because these results are seemingly conflicting, there is no evident point of view about the ability to use serum CRP concentration as a marker of post-ICU prognosis. In addition to this, the aforementioned studies have been criticized for their small sample sizes [10, 12, 15] and the selection of a patient population with a relatively good prognosis [12, 14].

The primary objective in this study was to test the hypothesis that elevated CRP levels at ICU discharge are associated with adverse outcome (ICU readmission and in-hospital mortality) in patients with a prolonged ICU stay (>48 h) and an anticipated attributable death rate post-ICU.

## Methods

This is a single-center retrospective cohort study of critically ill patients admitted to the ICU of the Medical Center Leeuwarden (MCL) in the Netherlands. According to applicable laws, the need for informed consent was waived by the regional ethical committee (Regionale Toetsingscommissie Patientgebonden Onderzoek, RTPO), due to the strict observational design of this study, using a database setup with clinical features of patients admitted to the ICU over the past 10 years. The ICU of the MCL is a closed format department and has 18 intensive care beds and four high care beds. Both medical and surgical patients are admitted to the department, including cardiothoracic patients. Long-stay ICU patients are discharged on weekdays during office hours, with little to no discharges in out-of-office hours. Patients are discharged from the ICU, when the intensivist decides they no longer require ICU-specific treatment for organ dysfunction, and their condition appears stable enough to be managed with low-intensity nursing care.

Patients who were discharged alive from the ICU with an ICU length of stay (LOS) >48 h were included in the study. For patients with multiple ICU admissions, only the first admission was included. Exclusion criteria were patients younger than 18 years of age, missing CRP data at discharge or loss to follow-up due to transfer to another hospital. The database analyzed in this study included details of ICU admissions between 2009 and 2012. Clinical predictors analyzed included demographics (age and sex), comorbidities, diagnosis group at admission and type of admission. The clinical predictors analyzed included severity of illness APACHE II [4], standardized mortality rate (SMR), SOFA [5] and SAPS II [6], days of mechanical ventilation and use of renal replacement therapy (continuous veno-venous hemofiltration, CVVH). APACHE II, SAPS II score and SMR were determined on the day of admission. The SOFA was daily calculated during ICU stay. Also, the LOS in hospital before ICU admission, the patient’s admission source (from the emergency department, transfer from a ward, or from the operating room), LOS during the ICU period, the destination after ICU discharge (to the ward, to a nursing home or to home), LOS after primary ICU discharge and mortality or ICU readmission were recorded.

 Serum CRP and white cell count (WCC) were routinely measured every morning during the complete ICU stay. Serum CRP was measured by a latex-mediated immunoturbidimetric method in plasma heparin, using a modular analyser (Roche Diagnostics, Mannheim, Germany), and WCC was measured in dipotassium ethylenediamine tetraacetic acid (EDTA) blood with a hematologic analyser (Abbot Laboratories, Santa Clara, CA, USA). Body temperature was measured at least every 8 h via internal ear. We extracted the following data at ICU admission and on the day of discharge: CRP, WCC and body temperature.

Patients were divided in two predefined subgroups: ‘high CRP’ at ICU discharge: CRP ≥75 mg/L and ‘low CRP’ at ICU discharge: CRP <75 mg/L, according to previous reports [16]. The primary endpoint of this study was adverse outcome. We defined adverse outcome as readmission to the ICU, and/or death on the ward after ICU discharge. Readmission was defined by discharge to an area that provided a lower level of care followed by return to the same ICU within the same hospitalization period. Post-ICU death is defined by death after ICU discharge within the same hospitalization; this included patients who died during their readmission on the ICU. In a secondary analysis of unfavorable outcome, we excluded all planned ICU readmissions and patients who were discharged from the ICU with ‘do-not-resuscitate’ orders in combination with limitation of future therapeutic interventions, and subsequently died in ward.

For statistical analysis, the statistical package for social science (SPSS 16 for Windows; SPSS Inc, Chicago, IL, USA) was used. Using the Kolmogorov–Smirnov test, data were tested for normal distribution. For continuous variables, normal distributed data are presented as mean ± standard deviation (SD), non-normal distributed data as median with 25th and 75th percentiles (interquartile range, IQR). Categorical variables are presented as percentage (%). Baseline characteristics and laboratorial data of both groups (CRP ≥75 mg/L and CRP <75 mg/L) are compared using the nonparametric Mann–Whitney *U* test or unpaired *t* test. All statistics were two-tailed, and a *p* value <0.05 was considered statistically significant. A multivariate logistic regression was used to identify variables independently associated with adverse outcome. All factors associated with adverse outcome with a *p* value ≤0.25 in univariate analysis were included. The model was refined in a backward stepwise manner, excluding at each step the least significant of any variables not significant at *p* < 0.05. Therefore, variables were tested at nonlinearity of the logit and multicollinearity. Hosmer–Lemeshow goodness-of-fit tests were performed to access the calibration of the new model. Also, CRP and discrimination of the model were tested to produce receiver operating characteristic (ROC) curves, in accordance with Ray et al. [17]. The area under the curve (AUC) with 95 % confidence interval (CI) was calculated in prediction of patient’s adverse outcome. The optimal cutoff value of CRP was defined as the value associated with the highest sum of sensitivity and specificity −1 (Youden index).

## Results

**Fig. 1:**
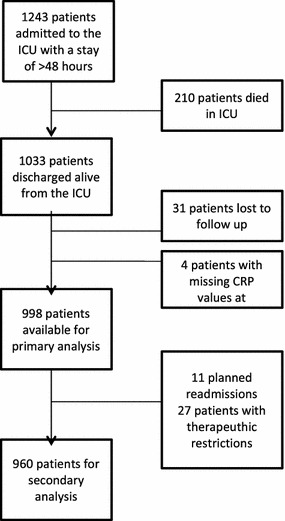
Flowchart

Out of 1239 patients with an ICU stay >48 h, a total of 1033 were discharged alive from the ICU between September 2009 and September 2012. Flowchart is shown in Fig. 1. After exclusions, 998 patients were available for primary analysis. Median age was 66 [65–74] and median APACHE II score 19 [15–24]. Baseline characteristics, type of admission and diagnostic subgroups are presented in Table 1. The overall in-hospital mortality rate was 5.5 % (*n* = 55), and the overall readmission rate was 9.5 % (*n* = 95). Out of 998 patients discharged alive from ICU, a total of 375 had CRP levels ≥75 mg/L (‘high CRP’) and 623 had CRP levels <75 mg/L (‘low CRP’). The ‘high CRP’ group, in comparison with the ‘low CRP’ group, had a significant lower SOFA score at ICU admission (6.7 ± 2.9 vs. 7.5 ± 2.9; *p* < 0.001), a lower APACHE II score at ICU admission (18 [14–22] vs. 19 [15–25]; *p* < 0.001) and a lower CRP value at ICU admission (57 [7–170] vs. 72 [15–180]; *p* = 0.046). Furthermore, these patients were more likely to be admitted for planned surgery (42 vs. 25 %; *p* < 0.001, Table 1). The ‘low CRP’ group was treated more often with renal support (17 vs. 9 %; *p* < 0.001) and had a longer LOS in ICU (9 [6–16] vs. 6 [4–8]; *p* < 0.001, Table 2). Patients in the ‘high CRP’ group had a readmission rate of 13.1 %. In the ‘low CRP’ group, this was 7.4 %; *p* = 0.003. The post-ICU mortality rate in the ‘high CRP’ group and ‘low CRP’ group was 6.9 % and 4.7 %, respectively; *p* = 0.127. The adverse outcome rate was significantly higher in the ‘high CRP’ group in comparison with the ‘low CRP’ group (17.9 vs. 10.1 %; *p* = 0.001). Hospital mortality in patients readmitted to the ICU was significantly higher than in the non-readmitted patients (20 vs. 4.3 %; *p* < 0.001). Eighty-five percentage of non-elective readmissions were related to the index admission. Median number of days to readmission was 3 [2–5] and not statistically different between groups (Table 3). In a secondary analysis, we furthermore excluded 11 electively readmitted patients (primarily for scheduled surgery) and 27 patients with therapeutic restriction orders. Outcomes variables are demonstrated in Table 4.

**Table 1:** Baseline characteristics

	All (*n* = 998)	‘High CRP’ (*n* = 375)	‘Low CRP’ (*n* = 623)	*p* value
Age (years)	66 [56–74]	66 [56–75]	66 [56–74]	0.349
Male sex (%)	62	61	62	0.924
LOS in hospital before ICU admission	1 [0–4]	1 [1–4]	1 [0–4]	0.655
APACHE II score admission	19 [15–24]	18 [14–22]	19 [15–25]	<0.001
SMR score admission	27 [12–53]	21 [10–41]	32 [14–57]	<0.001
SOFA score admission	7.2 (2.9)	6.7 (2.9)	7.5 (2.9)	<0.001
SAPS II score admission	32 [26–39]	31 [25–38]	33 [26–42]	0.048
CRP admission (mg/L)	66 [10–178]	57 [7–170)	72 [15–180]	0.046
WCC admission (×10^9^)	13.8 (8.2)	13.0 (6.9)	14.2 (8.9)	0.032
Temp admission (°C)	37.2 (1.2)	37.2 (1.1)	37.3 (1.3)	0.144
Comorbidities (%)				
Chronic renal failure	8	8	7	0.485
Hypertension	30	31	29	0.552
COPD	20	20	19	0.620
Diabetes mellitus	19	18	20	0.394
Chronic cardiovascular disease	18	18	18	0.781
Cancer	15	14	15	0.597
Other	6	6	7	0.534
Diagnosis group (%)				
Elective surgery	12	20	8	
Cardiac surgery	26	31	23	
Sepsis	38	32	41	
Trauma	2	2	2	
Cardiac arrest	8	7	9	<0.001
CFH	5	3	6	
COPD	2	1	2	
Intoxication	2	1	2	
Metabolic	1	1	2	
Others	4	3	5	
Type of admission (%)				
Medical	43	31	50	
Surgical—planned	31	42	25	<0.001
Surgical—urgent	26	27	25	
Patient’s origin (%)				
Emergency department	23	20	25	
Ward	19	12	23	<0.001
Operating room	54	66	47	
Other hospital	4	2	5	

Data are presented as mean ± SD (normal distribution), as median with interquartile range (non-normal distribution) or as % (categorical variable)

*CRP* C-reactive protein; high CRP, CRP ≥75 mg/L; low CRP, CRP <75 mg/L; *LOS* length of stay; APACHE II, Acute Physiology and Chronic Health Evaluation II; *SMR* standardized mortality rate; *SOFA* Sequential Organ Failure Assessment; *SAPS II* Simplified Acute Physiology Score II; *WCC* white cell counts; *Temp* body temperature; *COPD* chronic obstructive pulmonary disease; *CFH* congestive heart failure

**Table 2:** Primary and secondary outcomes

	All (*n* = 998)	‘High CRP’ (*n* = 375)	‘Low CRP’ (*n* = 623)	*p* value
Readmission (%)	9.5	13.1	7.4	0.003
In-hospital mortality (%)	5.5	6.9	4.7	0.127
Adverse outcome (%)	13.4	17.9	10.1	0.001
CRP discharge (mg/L)	58 [33–102]	115 [93–161]	39 [19–55]	<0.001
WCC discharge (×10^9^)	12.4 (5.7)	13.3 (5.7)	11.9 (5.6)	<0.001
Temp discharge (°C)	37.1 (1.3)	37.2 (1.1)	37.0 (1.4)	0.068
SOFA score discharge	3.0 (1.8)	3.3 (1.9)	2.9 (1.7)	<0.001
LOS ICU (days)	7 [5–12]	6 [4–8]	9 [6–16]	<0.001
Mechanical ventilation (%)	94	95	93	0.233
Days of mechanical ventilation (*n*)	3 [1–6]	2 [1–4]	4 [1–7]	<0.001
Renal support (%)	14	9	17	<0.001
Destination (%)				
Ward	96	99	95	
Home	1	0	1	0.004
Nursing/rehabilitation center	3	1	4	
LOS hospital after ICU discharge (days)	9 [5–16]	9 [6–16]	9 [5–16]	0.404

Data are presented as mean ± SD (normal distribution), as median with interquartile range (non-normal distribution) or as % (categorical variable)

*CRP* C-reactive protein; high CRP, CRP ≥75 mg/L; low CRP, CRP <75 mg/L; adverse outcome, intensive care unit readmission and/or in-hospital mortality; *WCC* white cell counts; *Temp* body temperature; *SOFA* Sequential Organ Failure Assessment; *LOS* length of stay; *ICU* intensive care unit

**Table 3:** Readmission

	All (*n* = 95)	‘High CRP’ (*n* = 49)	‘Low CRP’ (*n* = 46)	*p* value
Reason for readmission (%)				
Sepsis	4	2	2	
Non-sepsis	4.5	2	2	0.902
Elective	1	0.5	0.5	
Days between discharge and readmission (*n*)	3 [2–5]	3 [2–5]	3 [3–6]	0.785

Data are presented as median with interquartile range (non-normal distribution) or as % (categorical variable)

*CRP* C-reactive protein; high CRP, CRP ≥75 mg/L; low CRP, CRP <75 mg/L

**Table 4:** Outcome after exclusion ‘planned ICU readmissions’ and ‘restriction orders’

	All (*n* = 961)	‘High CRP’ (*n* = 358)	‘Low CRP’ (*n* = 602)	*p* value
Readmission (%)	8.7	12.3	6.6	0.003
In-hospital mortality (%)	2.9	3.6	2.5	0.308
Adverse outcome (%)	10.1	14	7.8	0.002

Data are presented as  %

*ICU* intensive care unit; *CRP* C-reactive protein; high CRP, CRP ≥75 mg/L; low CRP, CRP <75 mg/L; adverse outcome, intensive care unit readmission and/or in-hospital mortality

In a univariate analysis of all patients, female sex, age, APACHE II score, type of admission, CRP group (≥75 mg/L), temperature at discharge, SOFA score at discharge, LOS in ICU and use of renal support were factors associated with adverse outcome and included for further analysis. Multivariate logistic regression analysis showed that age, temperature at discharge, SOFA score at discharge, medical admission type, female sex and CRP ≥75 mg/L at discharge were significantly associated with adverse outcome (Table 5).

**Table 5:** Multivariate analysis of factors associated with adverse outcome

	Odds ratio [95 % CI]	*p* value
Age	1.02 [1.01–1.03]	0.018
Temp at discharge	1.59 [1.05–2.41]	0.028
SOFA score at discharge	1.21 [1.10–1.34]	<0.001
Medical admission	1.75 [1.18–2.58]	0.005
Female sex	1.50 [1.03–2.19]	0.035
CRP at discharge ≥75 mg/L	1.69 [1.14–2.50]	0.009

Hosmer–Lemeshow goodness-of-fit test: Chi-square, 14.4; *p* = 0.07

Adverse outcome, intensive care unit readmission and/or in-hospital mortality

*Temp* body temperature, *SOFA* Sequential Organ Failure Assessment, *CRP* C-reactive protein

**Fig. 2:**
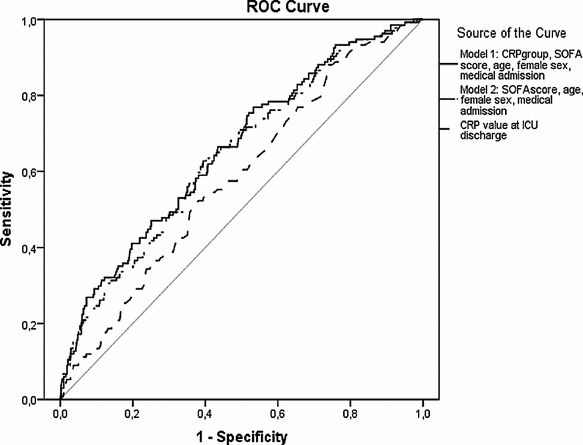
ROC curves of CRP concentrations at ICU discharge alone, CRP group combined with other covariates (model 1) and this model without CRP group (model 2), to discriminate between good outcome and adverse outcome after ICU discharge. CRP value at ICU discharge: area under the ROC curve: 0.58, 95 % CI 0.54–0.63; *p* = 0.002. Model 1: area under the ROC curve: 0.66, 95 % CI 0.61–0.71; *p* < 0.001. Model 2: area under the ROC curve: 0.65, 95 % CI 0.60–0.70; *p* < 0.001. *ROC* receiver operating characteristics, *CRP* C-reactive protein, *ICU* intensive care unit; adverse outcome: ICU readmission and/or in-hospital mortality, *SOFA* Sequential Organ Failure Assessment, *CI* confidence interval

In a post hoc analysis, a ROC curve was constructed for both CRP value at discharge, CRP group combined with other variables (model 1) and this model without CRP group (model 2). Results are shown in Fig. 2. The optimal cutoff (Youden index) for unfavorable outcome was at a CRP level of 72 mg/L, with a sensitivity of 57 % and a specificity of 62 %. Model 1 showed an area under curve of 0.66 (95 % CI 0.61–0.71; *p* < 0.001). Therewithal, the model was rather calibrated in both cohorts of patients (Hosmer–Lemeshow goodness-of-fit test: Chi-square, 14.4; *p* = 0.07).

## Discussion

In this retrospective cohort study with 998 patients discharged alive from the ICU and an ICU stay >48 h, we evaluated the relation between CRP levels at ICU discharge and ICU readmission and post-ICU mortality. Our data demonstrate that adverse outcome (ICU readmission and/or post-ICU mortality) was significantly increased in the ‘high CRP’ (CRP ≥75 mg/L) group in comparison with ‘low CRP’ (CRP <75 mg/L) group. This effect was particularly seen in relation to an increased risk of ICU readmission. A ‘high CRP’ at discharge was not significant related with post-ICU mortality alone. However, readmission to the ICU was associated with an almost fivefold increase in in-hospital mortality. These results were similar after excluding planned readmissions and patients with therapeutic restriction orders, who subsequently died in the hospital ward. A potential explanation could be the previous-described association between a state of persistent inflammation in survivors of an acute infection and deterioration of other diseases, such as cardiovascular disease, thus increasing long-term mortality [18]. However, our study was not designed to explore a potential causative relationship between (persistent) inflammation during critical illness and adverse outcome.

Although significant, CRP values at discharge would represent only a poor risk factor in the prediction of adverse outcome, with an area under the ROC curve of 0.58. Subsequent inclusion of age, female sex, SOFA score at discharge, temperature at discharge and medical admission type to a multivariate logistic regression analysis-based model provided a better predictor of adverse outcome. However, after excluding CRP value at discharge from this model, there was no significant difference in AUC, which means that adding CRP value to this predictive model poorly improves the ability to predict adverse outcome. It is of note that the APACHE II score at admission was excluded from the logistic regression analysis, despite its well-known overall predictive value for ICU and hospital mortality. This underlines the need for predictive models at ICU discharge that specifically take ICU-derived variables into consideration, such as the impact of therapy (e.g., renal replacement) and the patient response to the primary insult and therapy (e.g., CRP). This is also reflected by the fact that patients, discharged with a ‘high CRP,’ had lower CRP values and lower severity of illness scores at ICU admission, as compared to patients discharged with a ‘low CRP.’ Moreover, these patients had received less intense ICU care (days of mechanical ventilation and renal replacement therapy) and a shorter ICU stay. This may reflect the importance of ICU-acquired risk factors and a different inflammatory response in this specific patient group. Alternatively, the higher CRP values are a marker of earlier ICU discharge in comparison with patients who are admitted seemingly more ill.

Important differences of our data with previous studies are the selection of patients with a prolonged ICU stay (>48 h), with a concomitant higher likelihood of adverse. Secondly, we defined a composite primary outcome measure, consisting of both in-hospital mortality and ICU readmission. Finally, we a priori divided our groups into ‘high CRP’ and ‘low CRP,’ instead of outcome variables. Based on previous studies, we defined the CRP cutoff point at 75 mg/L. Post hoc the Youden index of the ROC curve showed a CRP level of 72 mg/L as the optimal cutoff point for adverse outcome, thus confirming our assumptions. However, this cutoff point has a weak sensitivity and specificity. Silvestre et al. [15] divided groups based on CRP value. However, this was a post hoc analysis after a primary analysis based on outcome. In accordance with our data, no correlation was found between CRP at discharge and post-ICU mortality, but no link was made between CRP and ICU readmission. Furthermore, their sample size was more than sixfold smaller than ours. In accordance with our study, Al Subaie et al. [14] investigated discharge CRP values and adverse outcome: readmission and in-hospital mortality, separately and together. All independent groups did not show a significant difference between discharge CRP and adverse outcome, in contrast to our results. Sample sizes of both studies were similar (1185 vs. 998), and the unplanned readmission rate in the paper of Al Subaie et al. was comparable (7 vs. 8.7 %) with identical unexpected death rates (2.9 %). However, median LOS ICU (1.8 days), APACHE II scores (16) and percentage of patients on mechanical ventilation (51 %) were considerably lower to our data, suggesting a significantly different patient population.

Our finding that discharge CRP was not associated with in-hospital mortality alone does not seem to be in line with the results of Ho et al. [11]. They found in their study with 603 patients that CRP at ICU discharge independently predicted subsequent death in hospital. Their study population was similar in its mixed surgical/medical nature, but CRP values were not available for all patients (only 73 % in non-survivors and 71 % in survivors), since this was not part of the standard measurements. Such non-random missing data create the possibility of a selection bias.

This study has several limitations. Firstly, this was a retrospective and single-center analysis, thus subjecting to biases. We do not know whether our findings can be extrapolated to other ICUs. Particularly, the specific logistics of ICU discharge in our hospital may be of critical importance in this respect. Secondly, by nature of our study design, a cause–effect relationship between a pro-inflammatory status and adverse outcome cannot be established. Despite elimination of many confounders by means of multivariate logistic regression analysis, the possibility of elevated CRP concentrations as a marker of a different unknown process cannot be excluded. In this respect, it is also conceivable that the routine use of daily CRP measurements in our ICU might have influenced clinical decision-making, including ICU discharge. Thirdly, a mixed group of medical and surgical patients were included; whether CRP will have a different performance in a particular subgroup of patients deserves further investigation. Finally, the AUC of both CRP level and the constructed model do not allow clinicians to make decisions with respect to ICU discharge on an individual patient bases. However, the presence of a combination of risk factors, as used in our model, may cause awareness of doctors for attributable morbidity and mortality post-ICU in individual patients. Whether interventions, such as postponement of ICU discharge or additional diagnostic/therapeutic procedures may reduce unfavorable outcome, remains to be established in future studies.

## Conclusions

In conclusion, this large retrospective cohort of critically ill patients (ICU LOS >48 h) in a mixed medical/surgical ICU substantiated the hypothesis that elevated CRP levels at ICU discharge are associated with readmission and in-hospital mortality. However, CRP at discharge represents only a very moderate risk factor and may not be used for individual clinical decision-making.
